# μ-Azido-κ^2^ *N* ^1^:*N* ^1^-μ-chlorido-bis­[(2-chloro-3-dimethyl­amino-1-phenyl­prop-1-en-1-yl-κ^2^ *C* ^1^,*N*)palladium(II)] chloro­form monosolvate

**DOI:** 10.1107/S1600536812047927

**Published:** 2012-12-08

**Authors:** Ana C. Mafud, Milene A. R. Oliviera, Maria T. P. Gambardella

**Affiliations:** a Instituto de Fisica de Sao Carlos, Av. do Trabalhador Saocarlense, 400 Sao Carlos, SP, Brazil; b Instituto de Quimica de Sao Carlos, Av. do Trabalhador Saocarlense, 400 Sao Carlos, SP, Brazil

## Abstract

In the binuclear title complex, [Pd_2_(C_11_H_13_ClN)_2_Cl(N_3_)]·CHCl_3_, each Pd^II^ atom has a slightly distorted square-planar geometry being coordinated by a C and an N atom of the 2-chloro-3-dimethyl­amino-1-phenyl­propyl ligand, a bridging Cl atom and an N atom of a bridging end-on azide group. There is a short intra­molecular C—H⋯Cl contact in the complex mol­ecule. In the crystal, the chloro­form solvent mol­ecule is linked to the complex *via* a C—H⋯π inter­action.

## Related literature

For the crystal structures of similar compounds, see: Moro *et al.* (2004 ▶); Caires *et al.* (2006 ▶). 
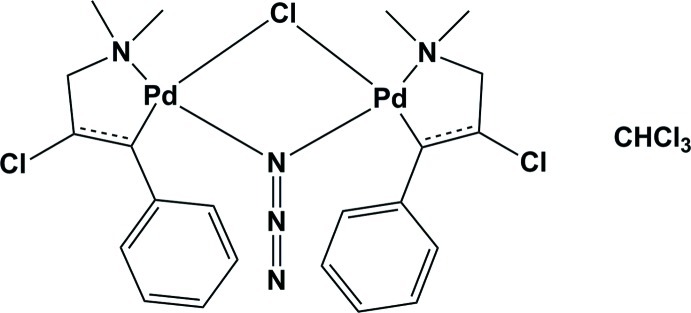


## Experimental

### 

#### Crystal data

[Pd_2_(C_11_H_13_ClN)_2_Cl(N_3_)]·CHCl_3_
*M* *_r_* = 799
Monoclinic, 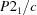

*a* = 15.416 (3) Å
*b* = 11.474 (3) Å
*c* = 17.094 (4) Å
β = 97.14 (2)°
*V* = 3000.2 (12) Å^3^
*Z* = 4
Mo *K*α radiation
μ = 1.76 mm^−1^
*T* = 293 K
0.2 × 0.1 × 0.1 mm

#### Data collection

Enraf–Nonius TurboCAD4 diffractometer
Absorption correction: ψ scan (North *et al.*, 1968 ▶) *T*_min_ = 0.691, *T*_max_ = 0.832
7536 measured reflections
7278 independent reflections
2686 reflections with *I* > 2σ(*I*)
*R*_int_ = 0.066
3 standard reflections every 120 min intensity decay: 1%

#### Refinement

*R*[*F* ^2^ > 2σ(*F* ^2^)] = 0.063
*wR*(*F* ^2^) = 0.170
*S* = 0.93
7278 reflections
329 parameters
H-atom parameters constrained
Δρ_max_ = 0.75 e Å^−3^
Δρ_min_ = −0.60 e Å^−3^

### 

Data collection: *CAD-4 EXPRESS* (Enraf–Nonius, 1994 ▶); cell refinement: *CAD-4 EXPRESS*; data reduction: *XCAD4* (Harms & Wocadlo, 1995 ▶); program(s) used to solve structure: *SHELXS97* (Sheldrick, 2008 ▶); program(s) used to refine structure: *SHELXL97* (Sheldrick, 2008 ▶); molecular graphics: *ORTEP-3 for Windows* (Farrugia, 2012 ▶); software used to prepare material for publication: *WinGX* (Farrugia, 2012 ▶).

## Supplementary Material

Crystal structure: contains datablock(s) I, global. DOI: 10.1107/S1600536812047927/su2528sup1.cif

Structure factors: contains datablock(s) I. DOI: 10.1107/S1600536812047927/su2528Isup2.hkl

Additional supplementary materials:  crystallographic information; 3D view; checkCIF report

## Figures and Tables

**Table 1:** Hydrogen-bond geometry (Å, °) *Cg*1 is the centroid of the C1*A*–C6*A* ring.

*D*—H⋯*A*	*D*—H	H⋯*A*	*D*⋯*A*	*D*—H⋯*A*
C11*A*—H11*B*⋯Cl1	0.96	2.74	3.299 (11)	118
C12—H12⋯*Cg*1^i^	0.98	2.77	3.713 (13)	163

Symmetry code: (i) 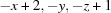.

**Fig. 1.**
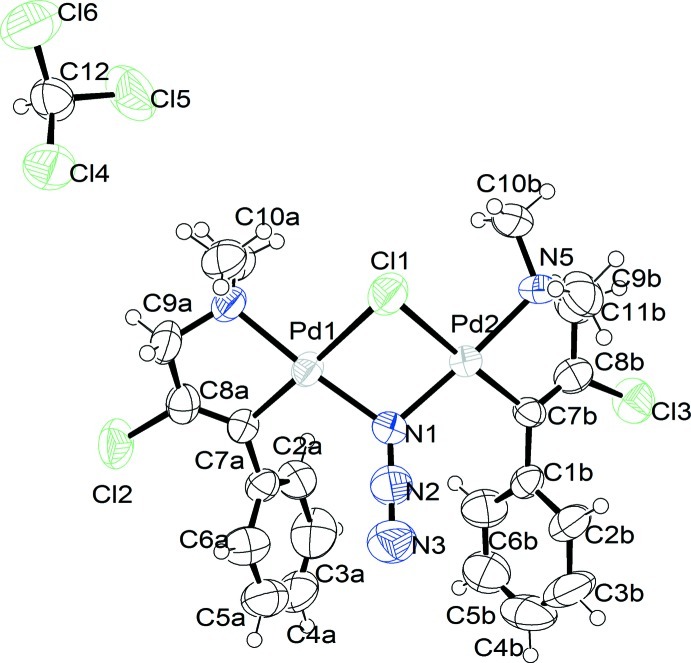
A view of the molecular structure of the title compound, with the atom numbering. The displacement ellipsoids are drawn at the 50% probability level.

## supplementary crystallographic information

### Comment

In the title compound, Fig. 1, the palladium atoms have distorted square planar geometry. Each palladium atom is connected to two N atoms. One is an N atom of the end-on bridging azide group [Pd1—N1 2.060 (7) and Pd2—N1 2.054 (8) Å] and the other one is the ligand amine group [Pd1—N4 2.064 (7) and Pd2—N5 2.086 (7) Å]. They are also connected to a C atom [Pd1—C7a 1.979 (9) and Pd2—C7b 1.988 (9) Å] and a bridging Cl atom [Pd1—Cl1 2.454 (3) and Pd2—Cl1 2.457 (3) Å]. These distances and angles are close to those reported for similar compounds (Moro *et al.*, 2004; Caires *et al.*, 2006). There is a short intramolecular C-H···Cl interaction (Table 1) in the complex. In the solid state, the title compound crystallized with one molecule of CHCl_3_.

In the crystal, the chloroform molecule is linked to the complex via a C-H···π interaction.

### Experimental

The title compound was synthesized from the interaction between the dimer [Pd(DMBA)(µX)]_2_ (where *X* = Cl, N_3_, NCO) and thiourea, being the product of the cleavage reaction. The reaction was performed by mixing a 1:2 stoichiometric amount of[Pd(DMBA)(µN_3_)]_2_ and thiourea in chloroform at room temperature with constant stirring during 1 h. After the reaction mixture was left for the solvent to slowly evaporate at room temperature. Large yellow needle-like crystals suitable for X-ray diffraction analysis were obtained.

### Refinement

The C-bound H-atoms were included in calculated positions and treated as riding atoms: C—H = 0.93, 0.96 and 0.97 Å, for CH, CH_3_ and CH_2_ H atoms, respectively, with *U*_iso_(H) = k × *U*_eq_(C) where k = 1.5 for CH_3_ H atoms, and = 1.2 for other H atoms.

### Crystal data

**Table:**

[Pd_2_(C_11_H_13_ClN)_2_Cl(N_3_)]·CHCl_3_	*F*(000) = 1576
*M**_r_* = 799	*D*_x_ = 1.769 Mg m^−^^3^
Monoclinic, *P*2_1_/*c*	Mo *K*α radiation, λ = 0.71073 Å
Hall symbol: -P 2ybc	Cell parameters from 15 reflections
*a* = 15.416 (3) Å	θ = 3.5–10.8°
*b* = 11.474 (3) Å	µ = 1.76 mm^−^^1^
*c* = 17.094 (4) Å	*T* = 293 K
β = 97.14 (2)°	Prism, red
*V* = 3000.2 (12) Å^3^	0.2 × 0.1 × 0.1 mm
*Z* = 4	

### Data collection

**Table:**

Enraf–Nonius TurboCAD4 diffractometer	2686 reflections with *I* > 2σ(*I*)
Radiation source: Enraf–Nonius FR590	*R*_int_ = 0.066
Graphite monochromator	θ_max_ = 28.1°, θ_min_ = 2.9°
non–profiled ω scans	*h* = −20→0
Absorption correction: ψ scan (North *et al.*, 1968)	*k* = 0→15
*T*_min_ = 0.691, *T*_max_ = 0.832	*l* = −22→22
7536 measured reflections	3 standard reflections every 120 min
7278 independent reflections	intensity decay: 1%

### Refinement

**Table:**

Refinement on *F*^2^	Primary atom site location: structure-invariant direct methods
Least-squares matrix: full	Secondary atom site location: difference Fourier map
*R*[*F*^2^ > 2σ(*F*^2^)] = 0.063	Hydrogen site location: inferred from neighbouring sites
*wR*(*F*^2^) = 0.170	H-atom parameters constrained
*S* = 0.93	*w* = 1/[σ^2^(*F*_o_^2^) + (0.0614*P*)^2^] where *P* = (*F*_o_^2^ + 2*F*_c_^2^)/3
7278 reflections	(Δ/σ)_max_ = 0.001
329 parameters	Δρ_max_ = 0.75 e Å^−^^3^
0 restraints	Δρ_min_ = −0.60 e Å^−^^3^

### Special details

**Table:**

Geometry. Bond distances, angles etc. have been calculated using the rounded fractional coordinates. All su's are estimated from the variances of the (full) variance-covariance matrix. The cell esds are taken into account in the estimation of distances, angles and torsion angles
Refinement. Refinement of *F*^2^ against ALL reflections. The weighted *R*-factor *wR* and goodness of fit *S* are based on *F*^2^, conventional *R*-factors *R* are based on *F*, with *F* set to zero for negative *F*^2^. The threshold expression of *F*^2^ > σ(*F*^2^) is used only for calculating *R*-factors(gt) *etc*. and is not relevant to the choice of reflections for refinement. *R*-factors based on *F*^2^ are statistically about twice as large as those based on *F*, and *R*- factors based on ALL data will be even larger.

### Fractional atomic coordinates and isotropic or equivalent isotropic displacement parameters (Å^2^)

**Table:**

	*x*	*y*	*z*	*U*_iso_*/*U*_eq_	
Pd1	0.74282 (5)	−0.05511 (7)	0.64046 (4)	0.0494 (3)	
Pd2	0.61088 (5)	0.04133 (7)	0.75721 (4)	0.0477 (3)	
Cl1	0.58807 (19)	0.0000 (3)	0.61517 (15)	0.0748 (11)	
Cl2	1.00891 (19)	−0.1565 (3)	0.58511 (18)	0.0912 (14)	
Cl3	0.5788 (2)	0.2278 (3)	0.98230 (18)	0.0847 (11)	
N1	0.7132 (5)	−0.0720 (8)	0.7541 (5)	0.060 (3)	
N2	0.7335 (7)	−0.1516 (10)	0.7953 (6)	0.072 (4)	
N3	0.7551 (7)	−0.2286 (10)	0.8356 (7)	0.097 (5)	
N4	0.7701 (6)	−0.0231 (7)	0.5275 (4)	0.056 (3)	
N5	0.5100 (6)	0.1623 (7)	0.7548 (5)	0.056 (3)	
C1A	0.9152 (6)	−0.1204 (9)	0.7375 (6)	0.052 (4)	
C1B	0.6717 (7)	0.0011 (8)	0.9329 (5)	0.049 (3)	
C2A	0.9345 (6)	−0.0278 (10)	0.7880 (6)	0.067 (4)	
C2B	0.6278 (8)	−0.0717 (10)	0.9781 (6)	0.073 (5)	
C3A	0.9793 (8)	−0.0472 (15)	0.8636 (7)	0.098 (6)	
C3B	0.6714 (13)	−0.1406 (12)	1.0365 (7)	0.107 (7)	
C4A	1.0045 (9)	−0.1571 (17)	0.8864 (7)	0.100 (7)	
C4B	0.7603 (14)	−0.1320 (14)	1.0537 (8)	0.112 (8)	
C5A	0.9832 (8)	−0.2477 (13)	0.8361 (9)	0.089 (6)	
C5B	0.8043 (9)	−0.0626 (14)	1.0078 (9)	0.100 (6)	
C6A	0.9400 (8)	−0.2308 (11)	0.7619 (7)	0.077 (5)	
C6B	0.7623 (8)	0.0030 (11)	0.9476 (7)	0.082 (5)	
C7A	0.8672 (6)	−0.1010 (8)	0.6573 (5)	0.050 (3)	
C7B	0.6233 (6)	0.0737 (8)	0.8724 (5)	0.047 (3)	
C8A	0.9010 (7)	−0.1115 (9)	0.5911 (6)	0.058 (4)	
C8B	0.5814 (7)	0.1672 (10)	0.8879 (6)	0.061 (4)	
C9A	0.8470 (7)	−0.0941 (10)	0.5124 (5)	0.066 (4)	
C9B	0.5306 (7)	0.2395 (10)	0.8233 (6)	0.076 (5)	
C10A	0.7924 (8)	0.1028 (10)	0.5224 (7)	0.082 (5)	
C10B	0.4982 (8)	0.2349 (10)	0.6818 (6)	0.086 (5)	
C11A	0.6979 (7)	−0.0499 (11)	0.4648 (6)	0.080 (5)	
C11B	0.4299 (7)	0.0972 (10)	0.7599 (7)	0.081 (5)	
Cl4	0.8090 (3)	−0.0041 (3)	0.2711 (2)	0.1048 (16)	
Cl5	0.7771 (3)	0.2344 (3)	0.3010 (3)	0.131 (2)	
Cl6	0.7452 (3)	0.1444 (4)	0.1462 (2)	0.147 (2)	
C12	0.8118 (8)	0.1356 (10)	0.2348 (7)	0.082 (5)	
H9A1	0.88070	−0.05380	0.47650	0.0800*	
H9A2	0.82820	−0.16860	0.48940	0.0800*	
H2A	0.91790	0.04730	0.77210	0.0800*	
H2B	0.56700	−0.07460	0.96930	0.0880*	
H9B1	0.47730	0.26910	0.84080	0.0910*	
H3A	0.99170	0.01500	0.89810	0.1180*	
H3B	0.64050	−0.19280	1.06410	0.1290*	
H9B2	0.56550	0.30510	0.80970	0.0910*	
H4A	1.03600	−0.17020	0.93560	0.1200*	
H4B	0.78980	−0.17290	1.09580	0.1340*	
H5A	0.99830	−0.32300	0.85260	0.1070*	
H5B	0.86500	−0.05930	1.01760	0.1200*	
H6A	0.92760	−0.29390	0.72820	0.0920*	
H6B	0.79440	0.04870	0.91660	0.0980*	
H10A	0.80110	0.12150	0.46920	0.1220*	
H10B	0.84490	0.11890	0.55700	0.1220*	
H10C	0.74540	0.14900	0.53770	0.1220*	
H10D	0.47440	0.18770	0.63790	0.1290*	
H10E	0.55370	0.26580	0.67200	0.1290*	
H10F	0.45880	0.29790	0.68860	0.1290*	
H11A	0.71670	−0.03560	0.41420	0.1210*	
H11B	0.64860	−0.00120	0.47110	0.1210*	
H11C	0.68150	−0.13030	0.46850	0.1210*	
H11D	0.38130	0.15010	0.75590	0.1220*	
H11E	0.43420	0.05700	0.80940	0.1220*	
H11F	0.42130	0.04180	0.71760	0.1220*	
H12	0.87190	0.15450	0.22620	0.0990*	

### Atomic displacement parameters (Å^2^)

**Table:**

	*U*^11^	*U*^22^	*U*^33^	*U*^12^	*U*^13^	*U*^23^
Pd1	0.0517 (5)	0.0582 (5)	0.0388 (4)	−0.0015 (5)	0.0081 (3)	−0.0009 (4)
Pd2	0.0481 (5)	0.0501 (5)	0.0452 (4)	0.0042 (4)	0.0068 (3)	0.0059 (4)
Cl1	0.0710 (19)	0.097 (2)	0.0568 (16)	0.0006 (17)	0.0095 (14)	0.0031 (15)
Cl2	0.0608 (19)	0.135 (3)	0.082 (2)	0.008 (2)	0.0257 (16)	−0.008 (2)
Cl3	0.096 (2)	0.083 (2)	0.0741 (19)	0.0033 (19)	0.0066 (17)	−0.0234 (18)
N1	0.063 (6)	0.061 (7)	0.055 (5)	0.017 (5)	0.009 (4)	0.008 (5)
N2	0.078 (7)	0.062 (7)	0.078 (8)	−0.001 (6)	0.015 (6)	0.006 (6)
N3	0.099 (9)	0.082 (9)	0.113 (9)	0.000 (7)	0.021 (7)	0.025 (7)
N4	0.072 (6)	0.057 (6)	0.040 (4)	−0.001 (5)	0.011 (4)	0.001 (4)
N5	0.063 (6)	0.052 (6)	0.049 (5)	0.008 (5)	−0.005 (4)	0.011 (4)
C1A	0.054 (6)	0.050 (7)	0.054 (6)	−0.005 (5)	0.012 (5)	0.008 (5)
C1B	0.065 (7)	0.045 (6)	0.038 (5)	−0.004 (5)	0.010 (5)	−0.004 (4)
C2A	0.058 (7)	0.078 (9)	0.060 (7)	0.000 (6)	−0.010 (5)	−0.011 (6)
C2B	0.097 (9)	0.074 (9)	0.049 (6)	−0.004 (7)	0.013 (6)	0.012 (6)
C3A	0.079 (9)	0.139 (13)	0.070 (8)	−0.015 (10)	−0.017 (7)	−0.027 (10)
C3B	0.185 (17)	0.083 (10)	0.055 (8)	0.015 (12)	0.024 (10)	0.025 (7)
C4A	0.087 (10)	0.157 (16)	0.053 (8)	0.023 (11)	−0.003 (7)	0.016 (10)
C4B	0.171 (18)	0.097 (12)	0.056 (9)	0.053 (13)	−0.030 (11)	−0.006 (8)
C5A	0.078 (9)	0.094 (11)	0.095 (10)	−0.001 (8)	0.009 (8)	0.031 (9)
C5B	0.076 (9)	0.128 (13)	0.091 (10)	0.013 (10)	−0.011 (8)	0.013 (10)
C6A	0.072 (8)	0.075 (9)	0.080 (9)	−0.015 (7)	−0.005 (7)	0.014 (7)
C6B	0.077 (9)	0.092 (10)	0.071 (8)	0.000 (7)	−0.014 (7)	0.006 (7)
C7A	0.054 (6)	0.053 (6)	0.044 (5)	0.002 (5)	0.010 (5)	−0.001 (5)
C7B	0.037 (5)	0.049 (7)	0.054 (6)	0.003 (5)	0.003 (4)	0.009 (5)
C8A	0.060 (7)	0.053 (7)	0.065 (7)	−0.008 (6)	0.020 (6)	−0.004 (6)
C8B	0.065 (7)	0.061 (8)	0.055 (6)	−0.008 (6)	0.002 (6)	0.002 (6)
C9A	0.065 (7)	0.089 (9)	0.048 (6)	0.002 (6)	0.018 (6)	−0.010 (6)
C9B	0.072 (8)	0.081 (9)	0.074 (8)	0.010 (7)	0.003 (6)	−0.004 (7)
C10A	0.105 (10)	0.074 (9)	0.068 (8)	0.014 (8)	0.020 (7)	0.011 (7)
C10B	0.109 (10)	0.078 (9)	0.073 (8)	0.044 (8)	0.020 (7)	0.021 (7)
C11A	0.080 (8)	0.108 (10)	0.050 (6)	0.008 (8)	−0.005 (6)	0.004 (7)
C11B	0.049 (7)	0.101 (10)	0.091 (9)	0.011 (7)	0.002 (6)	−0.003 (8)
Cl4	0.135 (3)	0.073 (2)	0.108 (3)	0.008 (2)	0.022 (2)	0.015 (2)
Cl5	0.145 (4)	0.102 (3)	0.144 (4)	0.046 (3)	0.007 (3)	−0.038 (3)
Cl6	0.206 (5)	0.143 (4)	0.087 (3)	0.024 (4)	0.004 (3)	0.021 (3)
C12	0.087 (9)	0.066 (8)	0.097 (9)	0.007 (7)	0.022 (7)	0.011 (7)

### Geometric parameters (Å, º)

**Table:**

Pd1—Cl1	2.454 (3)	C5A—C6A	1.371 (19)
Pd1—N1	2.059 (8)	C5B—C6B	1.37 (2)
Pd1—N4	2.060 (7)	C7A—C8A	1.309 (14)
Pd1—C7A	1.975 (9)	C7B—C8B	1.297 (15)
Pd2—Cl1	2.456 (3)	C8A—C9A	1.505 (14)
Pd2—N1	2.050 (8)	C8B—C9B	1.519 (15)
Pd2—N5	2.081 (9)	C2A—H2A	0.9300
Pd2—C7B	1.990 (9)	C2B—H2B	0.9300
Cl2—C8A	1.757 (11)	C3A—H3A	0.9300
Cl3—C8B	1.762 (11)	C3B—H3B	0.9300
Cl4—C12	1.721 (12)	C4A—H4A	0.9300
Cl5—C12	1.733 (13)	C4B—H4B	0.9300
Cl6—C12	1.724 (13)	C5A—H5A	0.9300
N1—N2	1.172 (14)	C5B—H5B	0.9300
N2—N3	1.144 (16)	C6A—H6A	0.9300
N4—C9A	1.487 (14)	C6B—H6B	0.9300
N4—C10A	1.490 (14)	C9A—H9A1	0.9700
N4—C11A	1.478 (13)	C9A—H9A2	0.9700
N5—C9B	1.471 (14)	C9B—H9B2	0.9700
N5—C11B	1.455 (14)	C9B—H9B1	0.9700
N5—C10B	1.493 (14)	C10A—H10B	0.9600
C1A—C7A	1.491 (13)	C10A—H10A	0.9600
C1A—C6A	1.373 (16)	C10A—H10C	0.9600
C1A—C2A	1.378 (15)	C10B—H10D	0.9600
C1B—C2B	1.372 (15)	C10B—H10E	0.9600
C1B—C6B	1.388 (16)	C10B—H10F	0.9600
C1B—C7B	1.459 (13)	C11A—H11C	0.9600
C2A—C3A	1.405 (16)	C11A—H11A	0.9600
C2B—C3B	1.381 (18)	C11A—H11B	0.9600
C3A—C4A	1.36 (2)	C11B—H11D	0.9600
C3B—C4B	1.37 (3)	C11B—H11E	0.9600
C4A—C5A	1.36 (2)	C11B—H11F	0.9600
C4B—C5B	1.36 (2)	C12—H12	0.9800
			
Cl1—Pd1—N1	82.3 (2)	C3A—C2A—H2A	120.00
Cl1—Pd1—N4	95.5 (3)	C1B—C2B—H2B	119.00
Cl1—Pd1—C7A	178.2 (3)	C3B—C2B—H2B	119.00
N1—Pd1—N4	175.1 (3)	C2A—C3A—H3A	120.00
N1—Pd1—C7A	99.4 (3)	C4A—C3A—H3A	120.00
N4—Pd1—C7A	82.9 (4)	C2B—C3B—H3B	120.00
Cl1—Pd2—N1	82.4 (3)	C4B—C3B—H3B	120.00
Cl1—Pd2—N5	95.4 (2)	C3A—C4A—H4A	120.00
Cl1—Pd2—C7B	177.3 (3)	C5A—C4A—H4A	120.00
N1—Pd2—N5	176.4 (3)	C3B—C4B—H4B	121.00
N1—Pd2—C7B	99.5 (4)	C5B—C4B—H4B	121.00
N5—Pd2—C7B	82.8 (4)	C4A—C5A—H5A	119.00
Pd1—Cl1—Pd2	81.89 (9)	C6A—C5A—H5A	119.00
Pd1—N1—Pd2	103.1 (4)	C4B—C5B—H5B	119.00
Pd1—N1—N2	124.5 (8)	C6B—C5B—H5B	119.00
Pd2—N1—N2	128.9 (8)	C1A—C6A—H6A	120.00
N1—N2—N3	178.6 (12)	C5A—C6A—H6A	120.00
Pd1—N4—C9A	109.0 (5)	C1B—C6B—H6B	120.00
Pd1—N4—C10A	107.7 (6)	C5B—C6B—H6B	120.00
Pd1—N4—C11A	114.8 (7)	N4—C9A—H9A1	110.00
C9A—N4—C10A	109.0 (9)	N4—C9A—H9A2	110.00
C9A—N4—C11A	107.8 (7)	C8A—C9A—H9A1	110.00
C10A—N4—C11A	108.5 (8)	C8A—C9A—H9A2	110.00
Pd2—N5—C9B	107.4 (6)	H9A1—C9A—H9A2	109.00
Pd2—N5—C10B	113.7 (7)	N5—C9B—H9B1	110.00
Pd2—N5—C11B	107.1 (6)	N5—C9B—H9B2	110.00
C9B—N5—C10B	108.7 (8)	C8B—C9B—H9B1	110.00
C9B—N5—C11B	111.2 (8)	C8B—C9B—H9B2	110.00
C10B—N5—C11B	108.7 (9)	H9B1—C9B—H9B2	109.00
C2A—C1A—C6A	119.4 (10)	N4—C10A—H10A	109.00
C2A—C1A—C7A	120.3 (9)	N4—C10A—H10B	109.00
C6A—C1A—C7A	120.3 (9)	N4—C10A—H10C	109.00
C2B—C1B—C6B	117.6 (10)	H10A—C10A—H10B	110.00
C2B—C1B—C7B	120.2 (10)	H10A—C10A—H10C	109.00
C6B—C1B—C7B	122.3 (9)	H10B—C10A—H10C	109.00
C1A—C2A—C3A	119.8 (11)	N5—C10B—H10D	109.00
C1B—C2B—C3B	121.7 (13)	N5—C10B—H10E	109.00
C2A—C3A—C4A	120.0 (13)	N5—C10B—H10F	109.00
C2B—C3B—C4B	119.9 (14)	H10D—C10B—H10E	110.00
C3A—C4A—C5A	119.2 (12)	H10D—C10B—H10F	109.00
C3B—C4B—C5B	118.5 (14)	H10E—C10B—H10F	110.00
C4A—C5A—C6A	121.8 (14)	N4—C11A—H11A	109.00
C4B—C5B—C6B	122.2 (14)	N4—C11A—H11B	109.00
C1A—C6A—C5A	119.8 (12)	N4—C11A—H11C	109.00
C1B—C6B—C5B	119.9 (11)	H11A—C11A—H11B	109.00
Pd1—C7A—C1A	122.4 (6)	H11A—C11A—H11C	110.00
Pd1—C7A—C8A	112.5 (7)	H11B—C11A—H11C	109.00
C1A—C7A—C8A	125.1 (9)	N5—C11B—H11D	109.00
Pd2—C7B—C1B	125.0 (7)	N5—C11B—H11E	109.00
Pd2—C7B—C8B	111.6 (7)	N5—C11B—H11F	109.00
C1B—C7B—C8B	123.5 (9)	H11D—C11B—H11E	110.00
Cl2—C8A—C7A	124.0 (8)	H11D—C11B—H11F	109.00
Cl2—C8A—C9A	114.2 (7)	H11E—C11B—H11F	110.00
C7A—C8A—C9A	121.7 (10)	Cl4—C12—Cl5	110.5 (7)
Cl3—C8B—C7B	125.6 (8)	Cl4—C12—Cl6	109.4 (7)
Cl3—C8B—C9B	112.5 (8)	Cl5—C12—Cl6	109.7 (7)
C7B—C8B—C9B	121.9 (9)	Cl4—C12—H12	109.00
N4—C9A—C8A	106.4 (7)	Cl5—C12—H12	109.00
N5—C9B—C8B	106.9 (9)	Cl6—C12—H12	109.00
C1A—C2A—H2A	120.00		
			
N1—Pd1—Cl1—Pd2	21.6 (3)	C11B—N5—C9B—C8B	85.9 (10)
N4—Pd1—Cl1—Pd2	−153.9 (2)	C6A—C1A—C2A—C3A	0.1 (15)
Cl1—Pd1—N1—Pd2	−26.6 (3)	C7A—C1A—C2A—C3A	179.6 (9)
Cl1—Pd1—N1—N2	133.7 (9)	C2A—C1A—C6A—C5A	0.3 (17)
C7A—Pd1—N1—Pd2	154.1 (4)	C7A—C1A—C6A—C5A	−179.2 (10)
C7A—Pd1—N1—N2	−45.6 (10)	C2A—C1A—C7A—Pd1	−69.6 (11)
Cl1—Pd1—N4—C9A	−155.7 (6)	C2A—C1A—C7A—C8A	110.2 (12)
Cl1—Pd1—N4—C10A	86.2 (7)	C6A—C1A—C7A—Pd1	110.0 (10)
Cl1—Pd1—N4—C11A	−34.8 (7)	C6A—C1A—C7A—C8A	−70.3 (14)
C7A—Pd1—N4—C9A	23.4 (7)	C6B—C1B—C2B—C3B	0.3 (16)
C7A—Pd1—N4—C10A	−94.7 (7)	C7B—C1B—C2B—C3B	−179.2 (10)
C7A—Pd1—N4—C11A	144.4 (8)	C2B—C1B—C6B—C5B	−2.8 (17)
N1—Pd1—C7A—C1A	−7.9 (8)	C7B—C1B—C6B—C5B	176.7 (11)
N1—Pd1—C7A—C8A	172.3 (7)	C2B—C1B—C7B—Pd2	−103.3 (10)
N4—Pd1—C7A—C1A	167.7 (8)	C2B—C1B—C7B—C8B	75.4 (13)
N4—Pd1—C7A—C8A	−12.1 (7)	C6B—C1B—C7B—Pd2	77.3 (12)
N1—Pd2—Cl1—Pd1	−21.7 (3)	C6B—C1B—C7B—C8B	−104.1 (13)
N5—Pd2—Cl1—Pd1	155.4 (2)	C1A—C2A—C3A—C4A	0.8 (17)
Cl1—Pd2—N1—Pd1	26.6 (3)	C1B—C2B—C3B—C4B	4.0 (19)
Cl1—Pd2—N1—N2	−132.5 (10)	C2A—C3A—C4A—C5A	−2.1 (19)
C7B—Pd2—N1—Pd1	−155.4 (4)	C2B—C3B—C4B—C5B	−6 (2)
C7B—Pd2—N1—N2	45.5 (11)	C3A—C4A—C5A—C6A	3 (2)
Cl1—Pd2—N5—C9B	−155.6 (6)	C3B—C4B—C5B—C6B	3 (2)
Cl1—Pd2—N5—C10B	−35.3 (7)	C4A—C5A—C6A—C1A	−1.6 (19)
Cl1—Pd2—N5—C11B	84.8 (7)	C4B—C5B—C6B—C1B	1 (2)
C7B—Pd2—N5—C9B	26.5 (7)	Pd1—C7A—C8A—Cl2	−177.5 (6)
C7B—Pd2—N5—C10B	146.8 (7)	Pd1—C7A—C8A—C9A	−2.2 (13)
C7B—Pd2—N5—C11B	−93.1 (7)	C1A—C7A—C8A—Cl2	2.7 (15)
N1—Pd2—C7B—C1B	−18.8 (9)	C1A—C7A—C8A—C9A	178.0 (9)
N1—Pd2—C7B—C8B	162.4 (8)	Pd2—C7B—C8B—Cl3	−178.0 (6)
N5—Pd2—C7B—C1B	164.0 (9)	Pd2—C7B—C8B—C9B	−0.2 (13)
N5—Pd2—C7B—C8B	−14.8 (8)	C1B—C7B—C8B—Cl3	3.2 (16)
Pd1—N4—C9A—C8A	−28.3 (9)	C1B—C7B—C8B—C9B	−179.1 (10)
C10A—N4—C9A—C8A	88.9 (9)	Cl2—C8A—C9A—N4	−162.9 (7)
C11A—N4—C9A—C8A	−153.5 (8)	C7A—C8A—C9A—N4	21.4 (14)
Pd2—N5—C9B—C8B	−31.1 (9)	Cl3—C8B—C9B—N5	−159.5 (7)
C10B—N5—C9B—C8B	−154.5 (9)	C7B—C8B—C9B—N5	22.5 (14)

### Hydrogen-bond geometry (Å, º)

Cg1 is the centroid of the C1A–C6A ring.

**Table:**

*D*—H···*A*	*D*—H	H···*A*	*D*···*A*	*D*—H···*A*
C11*A*—H11*B*···Cl1	0.96	2.74	3.299 (11)	118
C12—H12···*Cg*1^i^	0.98	2.77	3.713 (13)	163

Symmetry code: (i) −*x*+2, −*y*, −*z*+1.

## References

[bb1] Caires, A. C. F., Mauro, A. E., Moro, A. C., de Oliveira Legendre, A. & Ananias, S. R. (2006). *Quím. Nova*, **29**, 750–754.

[bb2] Enraf–Nonius (1994). *CAD-4 EXPRESS* Enraf–Nonius, Delft, The Netherlands.

[bb3] Farrugia, L. J. (2012). *J. Appl. Cryst.* **45**, 849–854.

[bb4] Harms, K. & Wocadlo, S. (1995). *XCAD4* University of Marburg, Germany.

[bb5] Moro, A. C., Mauro, A. E. & Ananias, S. R. (2004). *Eclet. Quím.* **29**, 57–61.

[bb6] North, A. C. T., Phillips, D. C. & Mathews, F. S. (1968). *Acta Cryst.* A**24**, 351–359.

[bb7] Sheldrick, G. M. (2008). *Acta Cryst.* A**64**, 112–122. 10.1107/S0108767307043930 18156677

